# Editorial: Advances in Modeling and Control of Soft Robots

**DOI:** 10.3389/frobt.2021.706514

**Published:** 2021-05-21

**Authors:** Concepción Alicia Monje Micharet, Cecilia Laschi

**Affiliations:** ^1^ Robotics Lab, Universidad Carlos III de Madrid, Madrid, Spain; ^2^ Department of Mechanical Engineering, National University of Singapore, Singapore, Singapore

**Keywords:** soft robotics, modeling of soft robots, control of soft robots, soft actuators, soft robotics applications

The emerging field of soft robotics is nowadays looking at innovative ways to create and apply robotic technology in our lives. It is a relatively new domain in the field of robotics, but one that has a lot of potential to change how we relate with robots and also how they are used. In natural environments and human-centric operations, where safety and adaptability to uncertainty are fundamental requirements, soft robots may beneficially show these characteristics: they can conform to variable but sensitive environments, adaptively move, manipulate and grasp unknown objects varying in size and shape and their soft condition allows them to squeeze through confined spaces.

In addition to the many challenges and research achievements on the material side, actuation and sensing techniques, and fabrication technologies, the questions on how to model soft robots and how to control their movements are challenging scientifically and important from the application perspective. Classical control approaches in robotics are nonlinear-model-based. However, the highly complex and nonlinear models necessary for a soft robotic system make this approach a difficult task and therefore seem to come to a limit in the presence of soft robots. Therefore, other methods have been applied seemingly being more useful in this context, such as learning-based control algorithms, model-free approaches like bang-bang control, control algorithms motivated by neuroscience, or morphological computation. These methods add new perspectives to the well-known model-based approach.

Such research challenges and the current achievements in the field have been discussed by the soft robotics community in a second forum on this topic, at the second workshop on “Advances in Soft Robots Control”, held on November 4th, 2019, in Macau, China, during the 2019 IEEE/RSJ International Conference of Intelligent Robots and Systems (IROS 2019). The workshop wanted to answer questions like “Do we have to rethink the basic approach in robot control, which is model-based, when it comes to controlling soft robotic systems?” The papers collected in this issue come from that discussion and compose the pictures of the achievements presented, with extensions following the discussion and analysis done in that interactive context. They cover achievements from the theoretical modeling of soft robots to their control, up to specific application-driven developments.

A few works answer the workshop question by rethinking the modeling approaches and techniques. They address dynamic modeling and related control of soft robots, starting from the current approaches based on statics, or second-order dynamics, and model predictive control (MPC), using basic lumped-parameters. Thuruthel et al. show how the dynamic model of a soft robot can be reduced to first-order dynamical equation, thanks to high damping and low inertia, with minimal loss in accuracy. The work by Hyatt et al. demonstrates that online model adaptation is key in soft robot dynamic modeling and shows their results with a model predictive control. It is based on the widely adopted piecewise constant curvature (PCC) assumption and shows an adaptive behavior, thanks to a model reference adaptive control. Dutra Gollob et al. present a model for predicting the output force profile of their vacuum-powered soft actuators, that uses a simplified geometrical approach and the principle of virtual work. The paper by Suphapol Diteesawat et al. addresses the specific case of electro-ribbon actuators, promising in soft robotics and challenging for control, as they exhibit pull-in instability and a very narrow contraction range for feedforward control: small contraction below the pull-in voltage threshold, complete contraction above that. The authors can access intermediate steady-states, not accessible using traditional feed-forward control, with a time-varying voltage profile that starts above pull-in threshold but is reduced afterward.

Schiller et al. move the focus on the whole robot body. They control the gait of a multi-limb robot by closing the control loop in Cartesian space, under the assumption of constant curvature (CC) and by reducing the joint space dimension from nine to two, describing the robot velocity space, i.e., the walking speed and the rotational speed. Angelini et al. also take a higher view and introduce a hierarchical, two-level, control architecture that takes neuroscience findings to ensure natural movements in articulated soft robots, such as learning by repetition, anticipatory behavior, reactive re-planning. It combines the low level of dynamic inversion and trajectory tracking with the high level that manages the degree of freedom (DOF) redundancy, allowing to control the system through a reduced set of variables.

Another way to rethink the basic approach in robot control, as in the workshop question, is by moving from model-based to model-free approaches. In the work by Al-Ibadi et al., a neural network (NN) controller laid in parallel with a proportional controller (P) tracks the non-linear behavior (elongation and bending) of a pneumatic muscle actuator (PMA). The parallel neural network proportional (PNNP) controllers provide a high level of precision and fast-tracking control system.

Some other works address the workshop question by outlining the importance of sensing and showing its instrumental role in control. Ibrahim et al. add sensing (an inertial measurement unit (IMU) and pressure sensing) to their fiber-reinforced actuator to couple it with PCC modeling and close the control loop on pressure and chamber lengths. Rupert et al. also address sensing and propose methods for placing length sensors on a soft continuum robot joint and for configuration estimation, with drastic error reduction. Chen et al. instead propose a different viewpoint and show how we can use soft arm compliant behavior to gain useful information. They show how they can estimate external loads acting on their soft arm, using a static model and controller.

Modeling is also relevant for rethinking soft robot design and improve their overall performance and usability. Lee et al. propose a design methodology for soft grippers that are customized to grasp single dedicated objects. They propose a fabrication method that can rapidly customize and fabricate soft grippers, thanks to a simplified analytical model based on geometric approximations and pseudo-rigid-body modeling theory. Yoder et al. address the design of prosthetic limbs with the aim of more closely mimicking intact neuromuscular systems and improve the capabilities of prosthetic users. They evaluate the performance of a hydraulically amplified self-healing electrostatic (HASEL) soft actuator, by using a kinematic model of the prosthetic finger to inform the design of their improved Peano-HASEL actuator with the goal of increasing the fingertip pinch force of the prosthetic finger.

This collection of papers provides a useful insight on recent, diverse, approaches to soft robot modeling and control. It shows how important scientific questions are addressed by the lively and productive scientific community in this field and outlines the scientific challenges that are still open and provide interesting opportunity for further research and progress.

